# Generation of human ER chaperone BiP in yeast *Saccharomyces cerevisiae*

**DOI:** 10.1186/1475-2859-13-22

**Published:** 2014-02-11

**Authors:** Evaldas Čiplys, Agota Aučynaitė, Rimantas Slibinskas

**Affiliations:** 1 Vilnius University Institute of Biotechnology, V.A. Graiciuno 8, Vilnius LT-02241, Lithuania; 2 UAB Baltymas, V.A. Graiciuno 8-231, Vilnius LT-02241, Lithuania

## Abstract

**Background:**

Human BiP is traditionally regarded as a major endoplasmic reticulum (ER) chaperone performing a number of well-described functions in the ER. In recent years it was well established that this molecule can also be located in other cell organelles and compartments, on the cell surface or be secreted. Also novel functions were assigned to this protein. Importantly, BiP protein appears to be involved in cancer and rheumatoid arthritis progression, autoimmune inflammation and tissue damage, and thus could potentially be used for therapeutic purposes. In addition, a growing body of evidence indicates BiP as a new therapeutic target for the treatment of neurodegenerative diseases. Increasing importance of this protein and its involvement in critical human diseases demands new source of high quality native recombinant human BiP for further studies and potential application. Here we introduce yeast *Saccharomyces cerevisiae* as a host for the generation of human BiP protein.

**Results:**

Expression of a full-length human BiP precursor in *S. cerevisiae* resulted in a high-level secretion of mature recombinant protein into the culture medium. The newly discovered ability of the yeast cells to recognize, correctly process the native signal sequence of human BiP and secrete this protein into the growth media allowed simple one-step purification of highly pure recombinant BiP protein with yields reaching 10 mg/L. Data presented in this study shows that secreted recombinant human BiP possesses native amino acid sequence and structural integrity, is biologically active and without yeast-derived modifications. Strikingly, ATPase activity of yeast-derived human BiP protein exceeded the activity of *E. coli-*derived recombinant human BiP by a 3-fold.

**Conclusions:**

*S. cerevisiae* is able to correctly process and secrete human BiP protein. Consequently, resulting recombinant BiP protein corresponds accurately to native analogue. The ability to produce large quantities of native recombinant human BiP in yeast expression system should accelerate the analysis and application of this important protein.

## Background

Human BiP (immunoglobulin heavy chain-binding protein), also known as GRP78, is an essential ER resident Hsp70 family chaperone [1]. Its functions in the ER are thoroughly studied and well described (reviewed in [2]). BiP is a major chaperone and most abundant protein in the ER, it plays a role in protein transport into the ER, folding, assembly, export and degradation, signal transduction and calcium homeostasis. Therefore, BiP is a central regulator of ER homeostasis and essential for embryonic cell growth and pluripotent cell survival [3]. Over the last decade BiP protein has attracted even more attention due to its newly discovered functions, localization in the cell, involvement in important human diseases and potential therapeutic applications. Recently it was recognized that in specific cell types or when subjected to stress, BiP can be located in cell compartments outside the ER, including the cell surface, the cytoplasm, the mitochondria and the nucleus. Also secretion into the extracellular space, where it affects the cell growth and signalling, was observed (reviewed in [4]).

Particularly intriguing is BiP involvement in progression of critical human diseases, such as cancer and neurodegenerative disorders. Current studies have established that BiP plays crucial and pleiotropic role in cancer progression (reviewed in [3,5]). It is overexpressed and translocated onto the cell surface in most cancer cells, where it regulates cell survival and proliferation, tumor progression and angiogenesis, and protects cancer cells against the adverse hypoxic and nutrient-deprived microenvironment. Naturally, targeting BiP sensitizes cancer cells to therapy [6]. Furthermore, preferential expression of BiP on the surface of tumor cells but not in normal organs suggests that surface BiP can serve both as a target as well as a mediator for cancer-specific therapy [7]. Recently BiP was suggested as a therapeutic target for neurodegenerative disorders (reviewed in [8]). Most neurodegenerative disorders are characterized by activation of the UPR and altered expression and activity of BiP in ageing cells, raising the question whether the lack of BiP could be a predisposing factor for many neurodegenerative disorders. In some cases endogenous overexpression of BiP was shown to have anti-apoptotic and neuroprotective effects in mice and rat models [9-11]. Taking together, these discoveries change the paradigm on BiP functions and suggest novel therapeutic approaches targeting this protein.

For further research and studies of the potential application of this protein, high quality and widely affordable recombinant human BiP protein is needed. Currently, *Escherichia coli* is the host of choice for the production of recombinant human BiP protein using various purification techniques [12-16], but resulting protein is less active than native analogue [16] and yields are low [16,17]. To the best of our knowledge, this work is the first to report yeast *Saccharomyces cerevisiae* as a host for the generation of human BiP protein. In our previous study, we inserted different human ER chaperones into the yeast to facilitate impaired maturation of recombinant Measles virus hemagglutinin [18,19]. Even though considerable amount of recombinant human BiP was found to be localized in the yeast ER, subsequent studies revealed that the protein was also secreted outside the yeast cell, similarly to another human ER chaperone ERp57 [20]. Here we report how this discovery allows simple and cost-effective purification of large amounts of active human BiP. Our studies revealed that the secretion of human BiP chaperone by yeast cells is mediated by correctly processed native signal sequence of BiP protein. Analysis of the yeast-derived human BiP showed that this protein highly resembles its native analogue. ATPase assay demonstrated that yeast-secreted recombinant protein is 3-fold more active when compared to recombinant BiP purified from *E. coli.* In conclusion, yeast *S. cerevisiae* is an excellent host for the production of native recombinant BiP protein.

## Results and discussion

### Expression and purification of human BiP protein

**Figure 1:**
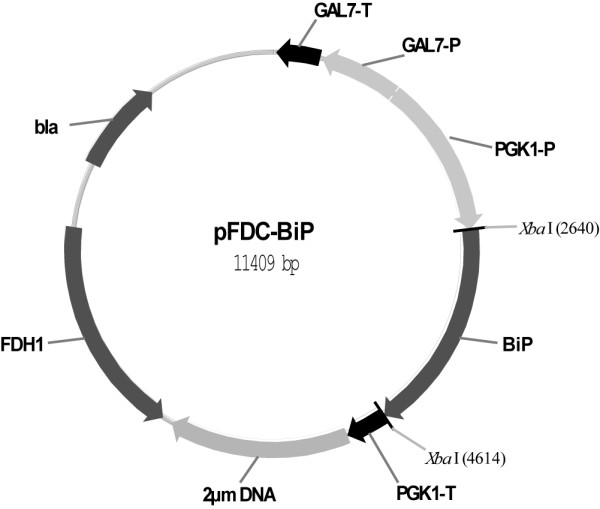
**Yeast vector pFDC-BiP for the expression of human BiP.** pFDC vector contains one inducible expression cassette under control of *Saccharomyces cerevisiae* GAL7 promoter with corresponding transcription terminator and the other cassette under control of constitutive *S. cerevisiae* promoter PGK1 with corresponding transcription terminator. 2 μm DNA, 1.74 kb fragment of yeast 2 μm DNA. PGK1-P, *PGK1* gene promoter ( - 1 to - 541 bp); PGK1-T, *PGK1* gene transcription terminator (371 bp). GAL7-P, *GAL7* gene promoter ( - 1 to - 716 nt); GAL7-T, GAL7 gene transcription terminator (381 bp); FDH1, *FDH1* gene of *Candida maltosa*, conferring resistance to formaldehyde; bla – beta lactamase gene, conferring resistance to ampicillin; BiP – human BiP protein coding gene (*HSPA5*, GenBank: AF216292).

**Figure 2:**
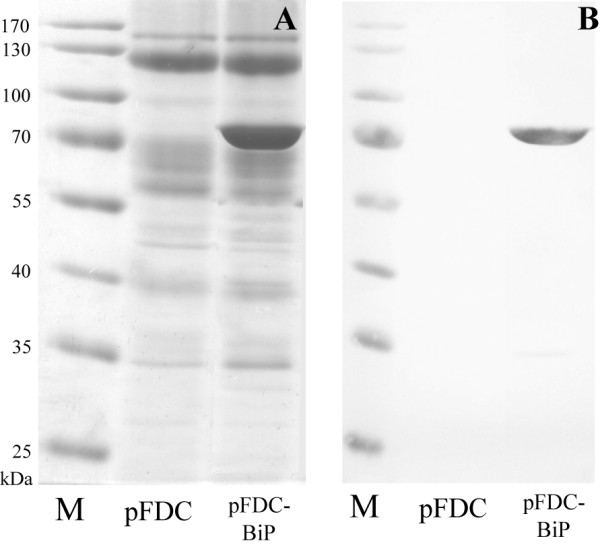
**Secretion of human BiP protein into the yeast growth medium.** SDS-PAGE **(A)** and Western blot against human BiP protein **(B)** of crude growth medium (*40*× concentrated) of yeast cells carrying control vector pFDC or pFDC-BiP for expression of human BiP. Cells were incubated for 36 h in YEPD medium. M – prestained molecular ladder (ThermoScientific, cat. no. 26618).

**Figure 3:**
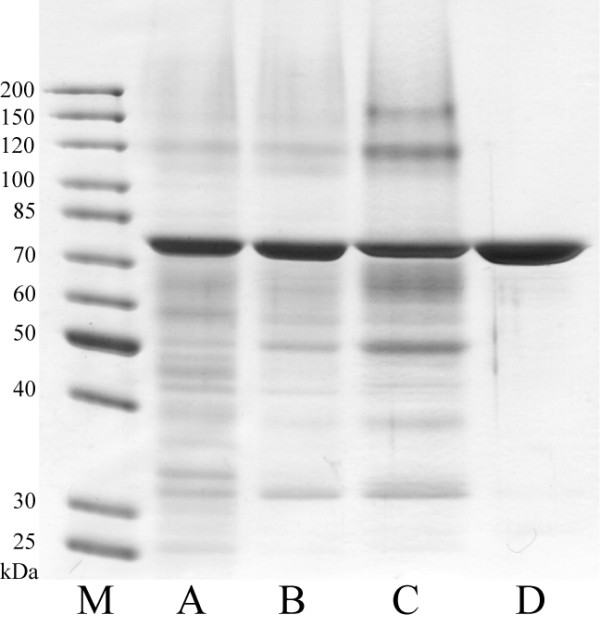
**Purification of secreted recombinant human BiP protein from yeast culture medium.** M – unstained protein ladder (ThermoScientific, cat. no. 26614). A – crude yeast growth medium (20× concentrated), B – yeast growth medium after microfiltration (20× concentrated), C – 20× concentrated proteins from yeast growth medium in binding buffer after tangential ultrafiltration; D – purified yeast-derived recombinant human BiP protein (5 μg).

Protein expression was carried out in *S. cerevisiae* transformants harboring multicopy autonomously replicating plasmid pFDC-BiP (Figure 1). In this study, we expressed the entire native amino acid sequence of human BiP precursor without any changes (i.e. yeast secretion signals and tags were not used). Yeast cells synthesized exactly the same polypeptide sequence as in human cells. Therefore, generated protein product can be considered as “native recombinant”. Surprisingly, recombinant human chaperone was not only found in membrane protein fractions of the yeast cells, as previously described [18], but was also found in fairly large amount in the growth medium (Figure 2). Secreted recombinant BiP protein was purified from the culture medium up to 95% purity as described in Methods (Figure 3). According to data obtained from densitometric analysis of SDS–PAGE gels, secreted human BiP protein constitutes for approx. 35-40% of all yeast secreted proteins (Figure 3 lane A), subsequent microfiltration increases its purity to approx. 50% (Figure 3 lane B) and one-step affinity chromatography using ATP-agarose is enough to reach 95% purity (Figure 3 lane D). Yields obtained were approx. 9–10 mg from 1 L culture medium with purification efficiency reaching up to 60%. It is a high yield compared to the production of this protein in bacterial expression system. Similar amount of recombinant mouse BiP was purified from 20 L of *E.coli* culture [17]. Purification of secreted human BiP was not as efficient as that of yeast-secreted human ERp57 protein [20], because BiP protein seems to bind to polyethersulfone membrane of tangential ultrafiltration cassettes. This is evident by the loss of about 1/3 of the protein after concentration by tangential ultrafiltration, where BiP constitutes to approx. 25% of total proteins (Figure 3 lane C). Despite this, secretion of human BiP into the yeast growth medium allows simple and cost-effective generation of native recombinant protein, which can be further optimized.

### Molecular weight, processing and oligomerization of recombinant human BiP protein

**Figure 4:**
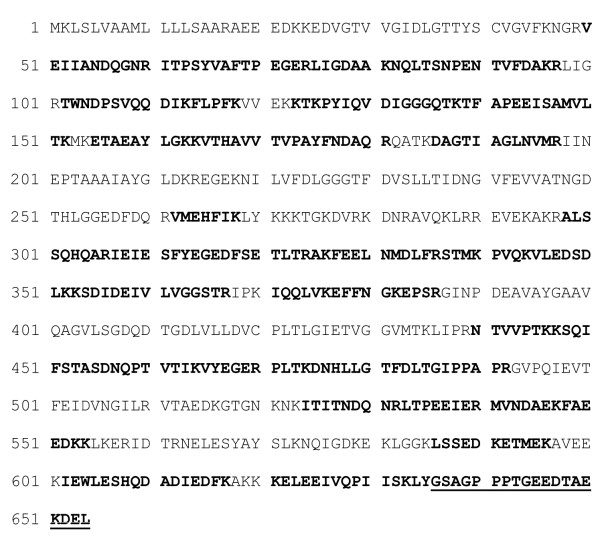
**Peptide mass fingerprinting of** *** S. cerevisiae* secreted GRP78/BiP protein by MALDI-TOF/TOF tandemic MS/MS together with UPLC/MS**^**E **^**method.**

**Figure 5:**
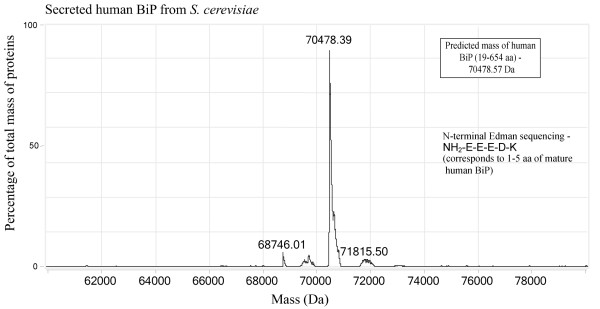
**ESI-MS of recombinant human BiP purified from *S. cerevisiae.***

The band of purified secreted BiP protein was excised from SDS-PAA gel and identified by trypsin digestion and MALDI-TOF/TOF tandemic MS/MS (mass spectrometry) together with UPLC/MS^E^ method using a service of the Proteomics Centre at the Institute of Biochemistry (Vilnius, Lithuania). Tryptic peptide mass fingerprinting confirmed that purified secreted protein represents human BiP, which was identified by both methods with a high level of confidence and ~57% sequence coverage (Figure 4). Figure 4 shows identified peptides (indicated in bold) of human BiP (Swiss-Prot:P11021). We were not able to identify N-terminal tryptic peptide by this method. However, search in a Swiss-Prot Protein Database using PLGS (Protein Lynx Global Service) search engine in UPLC/MS^E^ method identified a C-terminal human GRP78/BiP peptide (Y)GSAGPPPTGEEDTAEKDEL(-), which is underlined in Figure 4. This demonstrates that yeast-secreted human BiP protein possesses intact C-terminal amino acid sequence including KDEL ER retention/retrieval signal. Furthermore, N-terminal sequencing by Edman degradation confirmed that the first five N-terminal amino acids of the recombinant protein are NH_2_-EEEDK (Figure 5), which corresponds to the N-terminal sequence of mature human BiP protein after signal cleavage [21].

**Figure 6:**
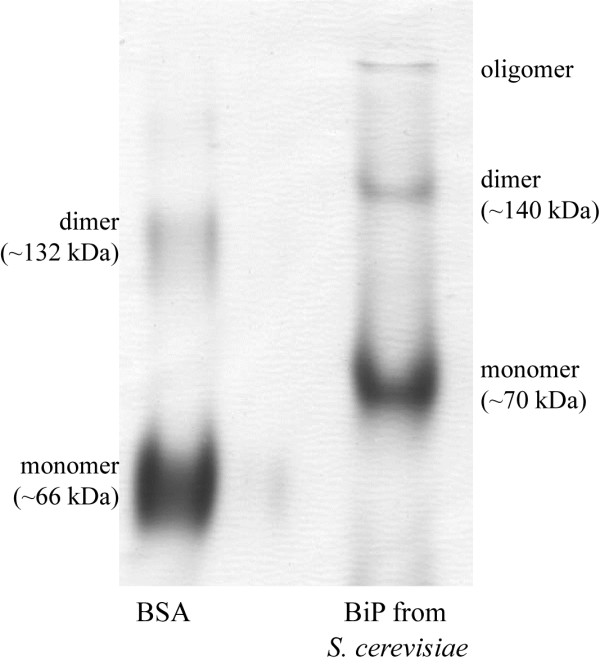
**Native PAGE of recombinant human BiP.** 5 μg of purified recombinant human BiP was loaded on gel. BSA was loaded as molecular weight marker.

The molecular mass of *S. cerevisiae*-secreted BiP protein was measured by electrospray mass spectrometry (ESI-MS) using Agilent Q-TOF 6520 mass spectrometer. ESI-MS of a whole *S. cerevisiae*-derived recombinant BiP protein molecule showed a molecular mass of 70478.39 Da, which exactly corresponds to theoretically predicted mass of mature human BiP (19–654 aa) polypeptide (Figure 5). It indicates that secreted recombinant BiP has no yeast-derived modifications, including phosphorylation and ADP-ribosylation. In mammalian cells free BiP can be reversibly phosphorylated and ADP-ribosylated. Such modifications lead to reversible aggregation and subsequent inactivation of BiP protein [22-26]. Therefore, BiP exists in mammalian cells as an active monomer, less active or inactive dimer, or in inactive oligomeric form [16,17,25]. To determine the oligomerization of the yeast-secreted human BiP, we analyzed it by native PAGE as described in Methods. As it was expected, unmodified yeast-secreted human BiP was present predominantly in monomeric form (Figure 6). For various applications, yeast-secreted recombinant protein might be even preferable over native protein, because majority of the recombinant BiP protein is in active (see below) monomeric form compared to diverse oligomerization of the native mammalian BiP protein.

Taken together, our data indicates that yeast-secreted recombinant human BiP exactly corresponds to the active monomeric form of native mature human BiP protein. It is correctly processed and does not carry any modifications or additional amino acid residues as it is often the case when using *S. cerevisiae* prepro α mating factor signal sequence for the secretion of recombinant proteins, because of the inefficient cleavage of Ste13 peptidase [27]. It also suggests that translocon machinery and signal peptide peptidase complex of yeast and human cells are compatible, because native signal sequence of human BiP protein is recognized and correctly cleaved in yeast cells. This allows translocation of recombinant protein into the ER followed by unexpected secretion outside the yeast cells. Secretion of human BiP protein by yeast is an interesting phenomenon, especially considering that signal sequence of BiP protein serves as a signal for translocation of protein into the ER, but not as a secretion signal. This finding not only allows simple generation of native human BiP protein in yeast cells, but also might serve as a convenient model to study mechanism of protein retention in the ER. As we reported earlier [20], human ERp57 protein is also secreted by yeast cells with the intact ER retention signal. Replacing ER retention signals of both BiP and ERp57 to yeast preferable ER retention signal HDEL [28] did not suppress the secretion of those proteins (our unpublished data). Also, overload of the yeast ER retrieval machinery as the reason for secretion of human ERp57 and BiP proteins can be omitted, because overexpression of yeast Kar2 protein with native HDEL ER retrieval sequence using the same pFDC vector did not lead to the secretion of this protein (Additional file 1: data 1). These results indicate that the retention of ER luminal proteins is complicated and still unsolved mechanism, which does not strictly depend only on HDEL/KDEL sequences, but is likely a combination of several factors. Observed secretion of human ER luminal proteins ERp57 and BiP by yeast cells can serve as a more convenient model to study this fundamental issue than the full genome-wide screening of yeast Kar2p secretion mutants [29].

### Conformation of yeast-secreted human BiP

**Figure 7:**
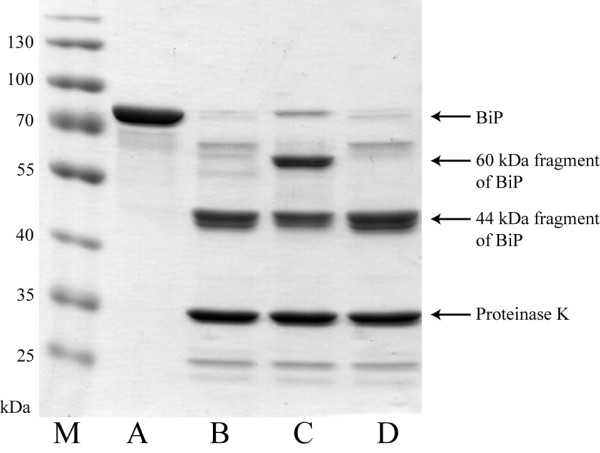
**Partial proteolysis of recombinant BiP with proteinase K in the presence of nucleotides.** M – prestained protein ladder (ThermoScientific, cat. no. 26616). Undigested recombinant BiP (A), or digested with proteinase K without nucleotides (B), in the presence of 100 μM ATP (C) or 100 μM ADP (D).

To assess the quality of yeast-derived human BiP protein, folding state and activity were determined. Correct folding was evaluated by partial proteolysis of recombinant human BiP protein. Partial protease digestion is often used as a measure of the structural integrity of BiP protein, because HSP70 proteins produce very distinctive proteolytic patterns when digested in the presence of nucleotides. ATP protects 60- and 44-kDa fragments from digestion, whereas ADP protects only the 44-kDa fragment [14,30]. *S. cerevisiae-*secreted human BiP protein was digested with proteinase K in the presence of ATP or ADP and analyzed by SDS-PAGE as described in Methods (Figure 7). Yeast derived human BiP protein bound ATP, and this protected a ~60 kDa fragment from proteolysis by proteinase K (Figure 7 lane C), whereas binding of ADP protected slightly larger amount of ~44 kDa fragment compared to the samples without added nucleotides or with added ATP (Figure 7 lane D compared to lanes B and C). Similar data was previously used to demonstrate correct folding of both native canine [30] and *E. coli*-expressed recombinant human BiP proteins [14].

### ATP-ase activity of yeast-derived human BiP protein

**Figure 8:**
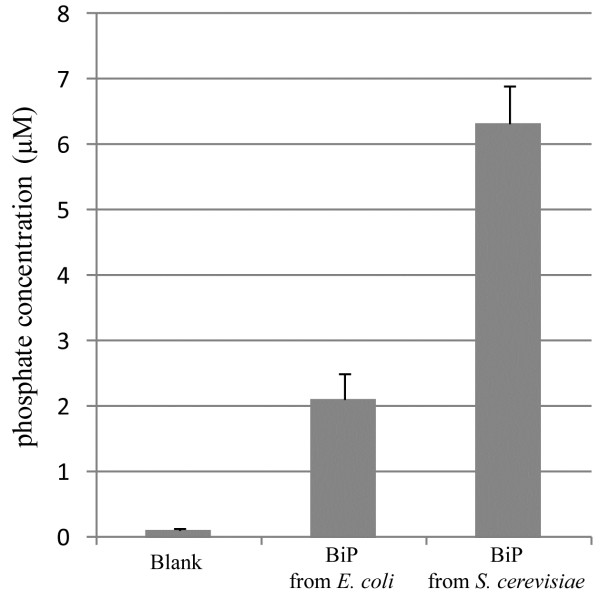
**ATPase activity of yeast-secreted human BiP.** The amount of released phosphate by 1 μg of either bacterial or yeast-derived human BiP was determined after incubation at 25°C for 75 min. with a non-radioactive procedure. Values are the mean of three separate experiments with an error bar representing SD.

ATPase activity of yeast-expressed human BiP protein was measured and compared with commercially available *E. coli-*derived human BiP using non-radioactive ATPase assay as described in Methods. This test is commonly used to assess activity of the BiP protein [12,31]. As it is evident in Figure 8, both proteins exhibited ATPase activity, but amount of liberated phosphate (μM/hr/μg protein) by yeast-derived BiP was 3-fold larger than that of bacteria-derived protein (~6.3 μM compared to ~2.1 μM) showing a 3-fold higher activity of the yeast-derived protein. The amount of phosphate liberated by *E. coli*-produced recombinant BiP in our experiments corresponds to that declared by manufacturer, validating our results. Such difference in activity between yeast- and bacteria-derived BiP proteins may be explained by the fact that yeast-secreted human BiP undergoes protein quality control throughout the yeast secretion pathway, which allows secretion of only correctly folded proteins. Meanwhile, *E. coli* does not have the ER, and the synthesis of recombinant human BiP is performed in different intracellular environment that may be less suitable for the proper maturation of the protein. Similar ratio of ATPase activity comparing native and *E. coli-*derived recombinant BiP protein was observed earlier [16], where recombinant hamster BiP protein showed only 30% activity of the native bovine liver BiP. Also, as we reported earlier [20], the yeast-secreted human ERp57 protein catalyzed the reduction of insulin in faster rate than recombinant human ERp57 from *E. coli*. Evidently higher activity of *S. cerevisiae-*secreted human BiP and ERp57 proteins compared to *E. coli*-derived recombinant analogues demonstrates that yeast is superior host for the production of BiP and other human ER chaperones.

### Secretion efficiency of human BiP in yeast

**Figure 9:**
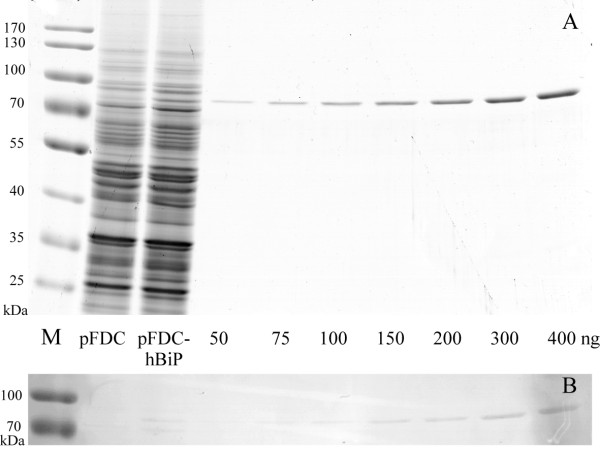
**Evaluation of amount of intracellular human BiP protein in yeast* S. cerevisiae* cells. (A)** SDS–PAGE of crude yeast lysates and indicated amounts of purified BiP; **(B)** Western blot using polyclonal antibodies against human BiP. M – prestained protein ladder (ThermoScientific, cat. no. 26618). pFDC and pFDC-hBiP – crude lysates (10 μg of whole cell protein in each lane) of yeast cells transformed with pFDC vector and pFDC-hBiP plasmid, respectively. 50, 75, 100, 150, 200, 300, 400 – amounts in nanograms of purified secreted human BiP protein loaded on gel.

As it was mentioned, human BiP protein was expressed previously in *S. cerevisiae* and was found inside the cell [18]. Here we have shown that this protein is also secreted. To evaluate the efficiency of secretion of human BiP protein driven by its native signal sequence, we compared amounts of intracellular and secreted BiP protein. SDS–PAGE analysis of crude lysates harbouring pFDC-BiP plasmid revealed clear additional band of recombinant human BiP compared to control cells carrying pFDC vector (Figure 9A, lanes pFDC and pFDC-hBiP). Also, quantitative Western blot using antibodies against human BiP protein was performed (Figure 9B). Densitometric analysis of both SDS-PAGE gel and Western blot showed that intracellular BiP constituted for approx. 0.9% of total cell protein. Evaluation of amount of proteins according to cell biomass produced from 1 L of culture revealed that approx. 70% of BiP was expressed internally (approx. 35 mg) and 30% was secreted into the culture medium (approx. 15 mg). Thus, secretion efficiency of human BiP protein in yeast *S. cerevisiae* cells is slightly higher than that of human ERp57 protein we reported earlier [20].

### Potential applicability of the results

This study revealed a new potential of the yeast to efficiently produce native recombinant human ER chaperones in the secreted form. It should be noted that a high-level secretion of these proteins by using their native signal sequences in yeast expression system is quite unusual. It is known that some secreted human proteins may be secreted by yeast cells using native secretion signal sequences [32,33], with a very few examples of a high-level secretion as in the case of human serum albumin in *P. pastoris*[33]. However, it may be expected that secreted human proteins will also be secreted in yeast cells using the same signal sequence. In contrast, the secretion of intracellular human proteins, such as ER-resident chaperones, is not expected when expressed in yeast. Moreover, the secretion level of human ER chaperones in yeast is unexpectedly high and allows efficient production of correctly processed recombinant products. As it was mentioned, in some cases the native human BiP was shown to be directed to the cell surface or secreted outside the cell where it is involved in a multitude of biological processes [4]. Possible therapeutic applications of BiP are mostly related to the extracellular protein form. For example, BiP is considered as a therapeutic agent of the third generation of biologics for immunological diseases such as rheumatoid arthritis [34]. It was shown that intravenously injected recombinant BiP is able to suppress arthritis and tissue inflammation in mice [35,36]. Yeast-secreted recombinant protein may be advantageous in such applications, because it is generated in the same way as the native extracellular BiP and corresponds to the native analog insofar as possible. Furthermore, yeast-derived heterologous proteins are free of toxic contaminations and are excellent tools for developing biopharmaceuticals, because *S. cerevisiae* is acknowledged as GRAS (generally regarded as safe) organism. Therefore, secretory expression of native recombinant human BiP in yeast could be exploited for efficient and safe production of potential therapeutic agent.

## Conclusions

Here we introduce the yeast *S. cerevisiae* as an excellent host for the generation of active human BiP protein. In this study we present evidence that yeast holds several key advantages over *E. coli* cells currently used for the synthesis of recombinant BiP protein: (a) newly discovered ability of yeast cells to recognize and process the native signal sequence of human BiP and their inability to retain it in the ER leads to secretion of this protein; (b) secretion of the protein allows simple and cost-effective one-step purification; (c) yeast-secreted human BiP exactly corresponds to the native protein; (d) yeast-derived human BiP is correctly folded and three-fold more active than recombinant BiP produced in *E. coli*. Considering the fact that growing amount of data associates BiP protein with critical human diseases and indicates this protein as a potential therapeutic target or agent, the ability of yeast cells to produce large amounts of native recombinant BiP protein might be invaluable. Also, secretion of this ER luminal human protein by yeast cells might serve as a convenient model to study retention of ER luminal proteins in the ER in general.

## Methods

### Construction of yeast vector for expression of human BiP

All DNA manipulations were performed according to standard procedures [37]. Recombinant plasmids were amplified in *E. coli* DH5αF’ cells. Human BiP coding gene (*HSPA5*, GenBank:AF216292) was cloned under the control of constitutive yeast *PGK1* gene promoter in pFDC vector, yielding pFDC-BiP plasmid (Figure 1), as it was described previously [18]. Briefly, cDNA encoding full-length human BiP protein precursor was amplified from commercial human adult liver cDNA library (Clontech) by PCR using specific oligonucleotide primers, digested with restriction endonuclease XbaI and cloned into yeast expression vector pFDC. Cloned *HSPA5* gene sequence was verified by DNA sequencing and generated plasmid pFDC-BiP was used for the transformation of yeast *S. cerevisiae* cells.

### Yeast strain, medium, transformation and cultivation

*S. cerevisiae* strain AH22 MATa leu2 his4 was used for expression experiments. Transformation of *S. cerevisiae* cells was performed by conventional LiCl method [37]. The selection of transformants resistant to formaldehyde was carried out on YEPD (yeast extract 1%, peptone 2%, dextrose 2%) agar supplemented with 4 mM formaldehyde. *S. cerevisiae* transformants were grown in YEPD medium supplemented with 4 mM formaldehyde.

### Protein expression and purification

Yeast cells carrying human *HSPA5* gene were grown for 36 h in YEPD medium. Cells were separated from the medium by centrifugation at 2000 g for 10 min. Yeast growth medium was further prefiltered through qualitative filter paper (VWR, cat. No. 516–0812) with subsequent microfiltration through filters with pore size of 1.6 μM (SartoriusStedim Biotech, cat. no. FT-3-1101-047), 0.45 μM (SartoriusStedim Biotech, cat. no. 15406–47) and 0.2 μM (SartoriusStedim Biotech, cat.n o. 15407-47-MIN). After microfiltration, proteins were concentrated and transferred into the binding buffer (20 mM HEPES, 50 mM NaCl, 10 mM MgCl_2_, pH 8.0) through tangential ultrafiltration using cassettes with 50 kDa cut-off membranes (SartoriusStedim Biotech, cat. no.VF20P3). Further, proteins were mixed with 6-AH-ATP-Agarose (Jena Bioscience, cat. no. AC-129 L) equilibrated in the same buffer and incubated for 2–3 hours at 4°C in batch format. Unbound proteins were removed by washing the resin with 20 column volumes of binding buffer while bound proteins were eluted with equal column volume of elution buffer (20 mM HEPES, 50 mM NaCl, 10 mM MgCl_2_, 5 mM ATP, pH 7.5). Elution fractions were analyzed by SDS-PAGE. Three subsequent elution fractions showed ~ 95% pure human GRP78/BiP protein. These fractions were pooled and dialysed against ATPase buffer (50 mM HEPES, 50 mM NaCl, 2 mM MgCl_2_, pH 6.8).

Preparation of crude yeast lysates, SDS-PAGE and Western blotting were performed exactly as described previously [18].

### Partial proteolysis of recombinant BiP in the presence of nucleotides

Partial proteolysis of yeast derived human BiP with proteinase K was performed as described by Wei and Hendershot [14]. 65-μl reactions were assembled that contained 10 μg of recombinant BiP, 2 μg of proteinase K (or similar volume of buffer for control), and 100 μM ATP or ADP in the ATPase buffer (50 mM HEPES, 50 mM NaCl, 2 mM MgCl_2_, pH 6.8). After incubation at 37°C for 25 min., the reaction was stopped by adding 10 μl of 1 mg/ml phenylmethylsulfonylfluoride and incubating it on ice for 30 min. The digested recombinant BiP was then analyzed by SDS-PAGE.

### ATPase assay

Non-radioactive ATPase assay was performed as described previously [12]. Reactions were performed in 50 μl volumes as follows: 1 μg of recombinant BiP protein (or equal volume of buffer for negative control) with 20 mM KCl and 20 μM ATP in ATPase buffer (50 mM HEPES, pH 6.8, 50 mM NaCl, 2 mM MgCl_2_) was incubated at 25°C for 75 min. Concentration of the phospate liberated from ATP was measured by spectrofotometer (TECAN Infinite 200, wave length 620 nm) using Malachite Green Phosphate Assay Kit (Cayman Chemical, cat. no. 10009325) according to manufacturers’ recommendations.

### Native PAGE

Purified recombinant BiP was mixed in equal volumes with sample buffer (0.01% Bromophenol Blue and 20% glycerol in TBE buffer (90 mM Tris, 90 mM Boric acid, 2 mM EDTA, pH 8)) and loaded onto 10% polyacrylamide gels. Gels were run in TBE buffer in 4°C at 100 V and 30 mA for 8–10 hours. After electrophoresis gels were stained with Coomassie brilliant blue R-250.

### Other methods and materials

N terminus sequencing of yeast secreted human BiP protein by Edman degradation was performed by AltaBioscience.

The molecular mass of protein was measured by electrospray mass spectrometry using Agilent Q-TOF 6520 mass spectrometer.

Protein concentrations were determined by Roti-Nanoquant Protein-assay (Carl Roth Gmbh., cat. no. K880).

Densitometric analysis of SDS-PAGE gels and Western blots, scanned with ImageSanner III (GE Healthcare) was performed with ImageQuant TL (GE Healthcare) software using default settings.

Precipitation of proteins from yeast growth medium for SDS-PAGE analysis was performed based on a defined methanol-chloroform-water mixture, as described earlier [38].

Recombinant human BiP protein purified from *E. coli* was purchased from StressMarq Biosciences Inc. (cat. no. SPR-107A).

Rabbit polyclonal antibodies against human BiP protein were purchased from Abcam (cat. no. ab21685).

## Competing interests

A patent application has been filed for the technology disclosed in this publication. EČ is a part-time employee of UAB Baltymas.

## Authors’ contributions

EČ was involved in all aspects of the experimental design, data collection, analysis and interpretation, and drafted the manuscript. AA performed protein purification, partial proteolysis and ATP assay experiments, and revised the manuscript. RS helped to design the experiment, analyzed data, reviewed and revised the manuscript. All authors read and approved the final manuscript.

## Supplementary Material

Additional file 1 **Concentrated culture medium of yeast cells over-expressing human BiP and Kar2 proteins.** M – prestained protein ladder (ThermoScientific, cat. no. 26616). 40× concentrated yeast growth medium of yeast cells harbouring control vector pFDC, vector pFDC-BiP for over-expression of human BiP and pFDC-KAR2 for over-expression of yeast Kar2 protein. Click here for file
