# Supplementary figures and images for: Generation of human ER chaperone BiP in yeast Saccharomyces cerevisiae
Source: Microb Cell Fact. 2014 Feb 11;13:22. doi: 10.1186/1475-2859-13-22 (PMC3926315; doi:10.1186/1475-2859-13-22)

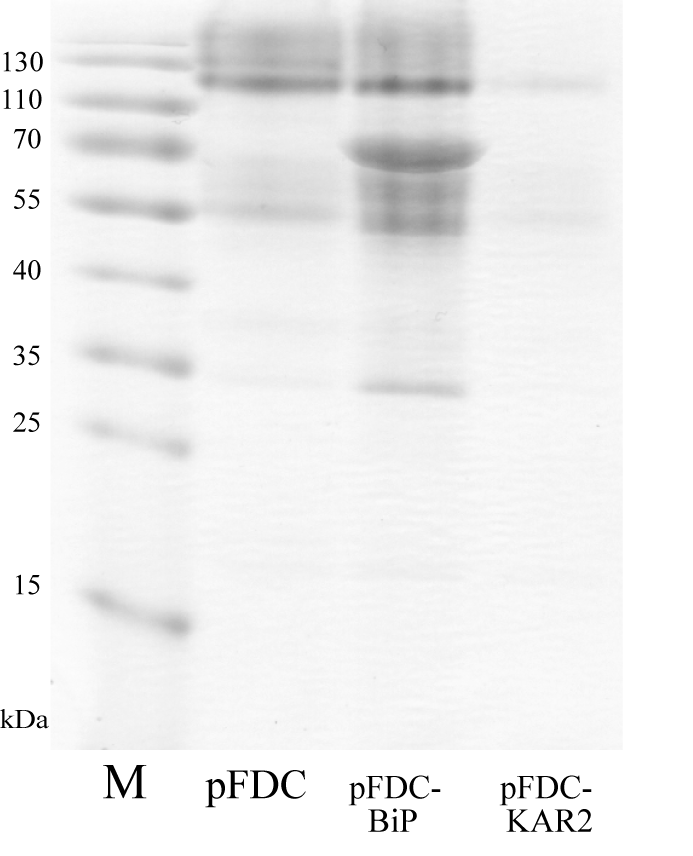

Supplement: Additional file 1 — Concentrated culture medium of yeast cells over-expressing human BiP and Kar2 proteins. M – prestained protein ladder (ThermoScientific, cat. no. 26616). 40× concentrated yeast growth medium of yeast cells harbouring control vector pFDC, vector pFDC-BiP for over-expression of human BiP and pFDC-KAR2 for over-expression of yeast Kar2 protein. [file 1475-2859-13-22-S1.tiff]
